# Window of opportunity clinical trial designs to study cancer metabolism

**DOI:** 10.1038/s41416-019-0621-4

**Published:** 2019-12-10

**Authors:** Francesca Aroldi, Simon R. Lord

**Affiliations:** Department of Oncology, Churchill Hospital, University of Oxford, Oxford, OX3 7LE UK

**Keywords:** Cancer metabolism, Cancer, Drug discovery

## Abstract

Window of opportunity trials exploit the ‘window’ of time after cancer diagnosis, typically prior to initiation of cancer therapy. In recent years this study design has become a more regular feature of drug development, as this ‘window’ provides an opportunity to carry out a thorough pharmacodynamic assessment of a therapy of interest in tumours that are unperturbed by prior treatment. Many of the first window trials interrogated the bioactivity of drugs being repurposed for cancer treatment, in particular the anti-mitochondrial agent, metformin. In this review, we describe examples of window study designs that have been used to assess drugs that target cancer metabolism with a focus on metformin. In addition, we discuss how window studies may aid the development of molecular metabolic cancer imaging.

## Background

Understanding the pharmacodynamic effects of cancer therapies early in the clinical drug development pathway is key to confirm target engagement, ensure appropriate selection of patients in future clinical trials and identify early resistance and potential drug combination. Window of opportunity trials allow a drug (or other intervention) of interest to be given to a patient over a short period of time, usually 2–-4 weeks, prior to the instigation of standard therapy. Biopsies of the tumour are typically carried out before and after drug alongside blood sampling to assay for circulating tumour markers, and functional tumour imaging may also be employed. Additionally, normal tissues may also be biopsied to assess for target engagement and blood sampling to profile patient specific features, for example for germline genetic alterations or host metabolic status.

Traditionally early phase cancer drug trials have only taken place in patients with heavily pre-treated tumours. Although detailed pharmacodynamic assessment is routinely carried out in these studies, drug activity will, at least in part, be determined by prior therapy and selection of resistant clones. While window studies may be carried out in this setting, this type of study design also gives an opportunity to profile the effects of treatment in tumours that are unperturbed by prior therapy, for example in the ‘window’ prior to curative surgery. In this setting comparison of samples taken for routine diagnostic purposes and at surgery may nullify the requirement to take extra tumour samples for research purposes.

Window studies should be considered a separate entity to Phase 0 trials. Phase 0 studies are designed to aid early drug development and generally used to resolve whether to take drug candidates forward into Phase 1 dose escalation studies. Typically, sub-therapeutic (micro-)doses of drug are given to patients to understand the agent’s biodistribution, assess for on-target effect and drug binding characteristics. Similar to window studies, Phase 0 trials will make use of paired biopsies to understand pharmacodynamic activity but there will also usually be an emphasis on pharmacokinetic profiling. In contrast, window studies are typically carried out at a later stage of drug development, using pharmacologic drug doses, and focus on understanding pharmacodynamic effect. Hence, this trial design is especially appropriate when investigating the bioactivity of drugs being repurposed for cancer treatment from other indications where the pharmacokinetic profile and safe dose level of the agent of interest is already well understood.

## Window study designs and cancer metabolism drug development

The concept of targeting cancer metabolism for therapeutic gain is decades old. Indeed, many of the older chemotherapy agents targeting nucleotide synthesis still used routinely in the clinic are known as ‘antimetabolites’ and in essence target cancer metabolism. These include the synthetic analogue of uracil, 5-fluorouracil, which as an inhibitor of thymidylate synthase hence limiting the synthesis of thymidine nucleotides for DNA synthesis. Methotrexate and pemetrexed are folate analogues that also inhibit nucleotide synthesis. Another anti-metabolite, gemcitabine, is a nucleoside analogue that is incorporated into DNA resulting in the inhibition of DNA polymerases. However, it is only much more recently that serious efforts have been made to develop a whole new class of drugs designed to target metabolic reprogramming in cancer cells. Most of these agents are small molecules that target a variety of different aspects of tumour metabolism, including glycolysis, amino acid (in particular glutamine metabolism), OXPHOS inhibitors and fatty acid synthesis. A number of these agents are now being tested in the clinic. Several oncogenes that play key roles in regulating metabolic pathways have also been successfully drugged–most notably members of the phosphoinositide 3-kinase–mammalian target of rapamycin (PI3K–mTOR) pathway. See Table 1 for a non-exhaustive list of therapies targeting cancer metabolism currently in the clinic.

**Table 1:** Selected agents that target tumour metabolism that are either approved or in clinical trials

Drug	Target	Metabolic pathway	Development phase
Methotrexate	Dihydrofolate reductase	Nucleotide metabolism	Approved cytotoxic chemotherapy agent
5-Fluorouracil	Thymidylate synthase	Nucleotide metabolism	Approved cytotoxic chemotherapy agent
Gemcitabine	DNA polymerases	Nucleotide metabolism	Approved cytotoxic chemotherapy agent
Metformin	Mitochondrial complex I	OXPHOS	Under investigation for repurposing
Phenformin	Mitochondrial complex I	OXPHOS	Under investigation for repurposing
Atovaquone	Mitochondrial complex III	OXPHOS	Under investigation for repurposing
IM156	Mitochondrial complex I	OXPHOS	Phase 1 trials
IACS-10759	Mitochondrial complex I	OXPHOS	Phase 1 trials
Ivosidenib	Mutant IDH1	TCA cycle metabolism	Approved for acute myeloid leukaemia
Enasidenib	Mutant IDH2	TCA cycle metabolism	Approved for acute myeloid leukaemia
AG-881	Mutant IDH1/2	TCA cycle metabolism	Phase 3 trials
FT-2102	Mutant IDH1	TCA cycle metabolism	Phase 2 trials
Devimistat	Pyruvate dehydrogenase/α-ketoglutarate dehydrogenase	TCA cycle metabolism	Phase 3 trials
CPI-613	PDK-1	TCA cycle metabolism	Phase 2 trials
CB-839	Glutaminase	Glutamine metabolism	Phase 2 trials
INCB001158	Arginase inhibitor: maintains arginine levels	Arginine metabolism	Phase 2 trials with checkpoint immunotherapy
Pegzilarginase	Arginine catabolism: depletes arginine levels	Arginine metabolism	Phase 1 trials
PEG-BCT-100	Arginine catabolism: depletes arginine levels	Arginine metabolism	Phase 1/2 trials
L-asparginase	Asparagine catabolism: depletes asparagine levels	Asparagine metabolism	Approved
AZD3965	Monocarboxylate transporter 1	Lactate metabolism	Phase 1 trials
TVB-2640	Fatty acid synthase	Lipid metabolism	Phase 2 trials

Window study designs have been widely utilised in recent years to profile the pharmacodynamic effects of therapies that target cancer metabolism. Much of the initial experience in this context stemmed from studies designed to tease out the anticancer effects of old drugs used for other indications but being repurposed for cancer treatment, in particular metformin. Other agents of interest for repurposing that target cancer metabolism include the fellow biguanide agent, phenformin, and also some anti-parasitic and lipidaemia drugs known to be mitochondrial toxins^1^ (also see Table 1).

Determination of target engagement is critical at an early stage of drug development. Most commonly, immunohistochemistry is employed to assay for the direct target in question or activation/inactivation of downstream pathways. RNASeq profiling is also now routinely used in drug development to identify mechanisms of early resistance and potential therapy combinations. Transcriptomic pathway analysis of samples pre- and post-drug can characterise metabolic response to drug therapy and evidence of target engagement. For drugs that target cancer metabolism, the use of functional imaging and metabolomic profiling may give additional insight into bioactivity. On-target effect using samples from surrogate tissues, for example skin biopsies, may be explored at least initially. However, very different pharmacodynamic effects may be seen in tumour cells with specific genetic alterations or in the context of the microenvironment of a hypoxic and poorly vascularised tumour, the latter especially relevant with regard to agents that target cancer metabolism.

Traditionally, in order to determine the dose and schedule of cancer drugs the emphasis has been to profile toxicity and pharmacokinetic parameters. However, there is now a realisation that it is necessary to have an understanding of how target engagement may vary in a population at different dose levels. Tabernero et al.^2^, designed an elegant study in which both tumour and skin biopsies were taken from 55 patients with metastatic renal carcinoma at baseline and after 4 weeks of the mTOR inhibitor, everolimus, using five different schedules/dose levels. Here, a schedule and dose-dependent inhibition of mTOR pathway signalling was observed using immunohistochemistry to assay for phosphorylation of several downstream members of this pathway—including protein S6 kinase 1 and eukaryotic initiation factor 4E (elF-4E) binding protein 1 (4E-BP1)—with consistency between tumour and skin biopsies. This work demonstrated that pharmacodynamic effects occurred below the maximum tolerated dose and also informed the dose and schedule taken forward for further study.^2^

## Window studies and metabolomic profiling

The development of the field of cancer metabolomic profiling has opened the door to these techniques as a novel assay to understand pharmacodynamic effect. 2-Hydroxyglutarate or 2-HG is an oncometabolite produced to strikingly high levels by tumours harbouring somatic isocitrate dehydrogenase mutations (IDH-1 or IDH-2), such that it can be detected in the peripheral blood of patients. There is therefore the potential to follow tumour response to either conventional or novel therapy using this marker. For example, enasidenib is a selective IDH2 inhibitor, which is in early phase clinical trials for patients with acute myeloid leukaemia, a disease which commonly harbours IDH-2 mutations. In one study as proof of target engagement there was a median 93% reduction in 2-HG levels following 4 weeks of treatment.^3^

Surgical window trials offer an opportunity to understand the metabolic fates within human tumours of particular carbon sources by infusing ^13^C-labelled substrates into patients just prior to tumour sampling at surgery. For example, Faubert et al infused ^13^C-lactate into study subjects undergoing surgery for non-small cell lung cancer and showed that these tumours utilise lactate over glucose to feed carbon into the TCA cycle.^4^ Fan et al. used ^13^C-glucose in lung cancer patients and identified increased pyruvate carboxylase activity compared to surrounding normal lung tissue.^5^ Using a similar approach Hensley et al. showed that there was significant intratumour heterogeneity with regard to substrate utilisation between different tumour areas.^6^ These techniques also have the potential to be applied to cancer metabolism drug studies to characterise in detail the effects of therapy on metabolic fluxes.

## Applying metabolic imaging in window studies

Modern molecular imaging already plays a central role in the diagnosis and staging of cancer patients, and to assess response to drug therapy. In the context of drug development molecular imaging has been commonly employed to assess drug distribution, target affinity and pharmacokinetics using radiolabelled drug. Additionally, positron emission tomography (PET) tracers have been developed to image drug target expression with a view to selecting patients for therapy and monitoring pharmacodynamic response. Examples include imaging human epidermal growth factor receptor 2 (HER2) using the tracer ^111^In-trastuzumab, vascular endothelial growth factor A (VEGFA) using ^89^Zr-bevacizumab^7^ and oestrogen receptor expression using [^18^F] fluoroestradiol (^18^F-FES).^8,9^ However, functional cancer imaging has long exploited the high metabolic requirements of tumour cells for clinical purposes and hence significant opportunity exists to use these techniques to image ‘cancer metabolism’. For example, PET with 2-deoxy-2-(fluorine-18)fluoro- D-glucose (^18^F-FDG PET), a radiolabelled analogue of glucose, is routinely used for cancer staging and evaluation of disease response to cancer therapy and within the context of drug trials. Although not available as part of standard clinical practice, use of dynamic ^18^F-FDG PET scans in research studies may be more sensitive in evaluating metabolic response to therapy and enable assessment of tracer distribution over time.^10^

While ^18^F-FDG PET-CT has been used in many early phase cancer drug trials to assess for metabolic response as an early indicator of clinical activity, other tracers that ‘image’ cancer metabolism have the potential to be used in pharmacodynamic studies to answer specific research questions. ^18^F-fluoromisonidazole (^18^F-MISO), a PET tracer that images hypoxia was used in a study of buparlisib, a class 1 PI3K inhibitor, to show that this drug could reduce hypoxia in non-small cell lung cancers with a view to its use as a sensitiser to radiotherapy.^11^ Uptake of the synthetic amino acid PET tracer, Fluciclovine (^18^F-FACBC), which is now widely used for the staging of prostate cancer, has been shown to be a marker for the high-affinity glutamine transporter, ASCT2.^12,13^ Recently, 3-^18^F-fluoro-2,2-dimethylpropionic acid (^18^F-FPIA) has been developed, the first PET tracer to image fatty acid oxidation and is now being evaluated in clinical studies.^14^ Choline is an essential substrate for phospholipid synthesis and activation of choline metabolism is associated with tumour progression^15,^ and acetate has been shown as a key substrate for lipid synthesis especially under conditions of metabolic stress.^16^ ^11^C-Choline and ^18^F-fluoroacetate are PET tracers that have shown potential for the staging of prostate cancer but also have potential use for imaging lipid metabolism in research studies.^17^ Technetium (^99^mTc) sestamibi is a long-established nuclear medicine agent predominantly used for cardiac imaging. However, it has been shown to be preferentially taken up into many tumour types and uptake corresponds to mitochondrial membrane potential.^18,19^ Please see Table 2 for a more comprehensive list of PET radiotracers that image cancer metabolism and are currently being evaluated or are in use in clinic.

**Table 2:** Selected PET radiotracers that image tumour metabolism that are either approved or available for clinical research

Tracer	Description	Metabolic pathway	Clinical indications
^18^F-Fluorodeoxyglucose (^18^F-FDG)	Radiolabelled glucose analogue	Glucose metabolism	Widespread application for cancer staging and on treatment monitoring
^18^F-Fluorodeoxygalactose (^18^F-FDGal)	Radiolabelled galactose analogue	Galactose metabolism	Research purposes only
^11^C-Choline	Radiolabelled choline	Phospholipid metabolism	Detection of prostate cancer recurrence
^18^F-Choline	Radiolabelled choline	Phospholipid metabolism	Staging of brain and bladder tumours
^11^C-Acetate	Radiolabelled acetate	Lipid metabolism	Detection of prostate cancer recurrence
3-^18^F-Fluoro-2,2-dimethylpropionic acid (^18^F-FPIA)	Radiolabelled carboxylic acid	Fatty acid oxidation	Research purposes only
^18^F-Fluorothymidine (^18^F-FLT)	Radiolabelled thymidine	Nucleotide metabolism	Lymphoma staging
^18^F-Fluoromisonidazole (^18^F-FMISO)	Radiolabelled nitroimidazole analogue	Hypoxia	Research purposes only
^18^F-Luoroazomycin arabinoside (^18^F-FAZA)	Radiolabelled nitroimidazole analogue	Hypoxia	Research purposes only
^64^Cu-Diacetyl-bis(N^4^-methylthiosemicarbazone) (^64^Cu-ATSM)	Diacetyl-bis(N^4^-methylthiosemicarbazone) radiolabelled with copper	Hypoxia	Research purposes only
^18^F-Fluciclovine (^18^F-FACBC)	Radiolabelled synthetic analogue of L-leucine	Amino acid metabolism	Detection of prostate cancer recurrence
^11^C-Methionine (^11^C-MET)	Radiolabelled methionine	Amino acid metabolism	Brain tumour diagnosis
^11^C-Glutamine	Radiolabelled glutamine	Amino acid metabolism	Research purposes only
(^18^F-Fluoropropyl)-*L*-glutamic acid (^18^F-FSPG)	Radiolabelled glutamate analogue	Amino acid metabolism	Research purposes only
^18^F-Fluoroethyltyrosine (^18^F-FET)	Radiolabelled L-tyrosine	Amino acid metabolism	Research purposes only
^18^F-Dihydroxyphenylalanine (^18^F-DOPA)	Radiolabelled phenylalanine	Dopamine metabolism	Neuroendocrine tumour staging

A number of magnetic resonance imaging (MRI) techniques have now also been developed that can image biomolecules of interest. These techniques have some advantages over PET imaging in that they are typically less invasive and avoid radiation exposure and hence are more suited to several measurements over a short period. Functional MRI and, in particular, chemical exchange saturation transfer (CEST) MRI measures the chemical exchange of protons between hydroxyl groups and water. For example, glucoCEST MRI has been shown to be a sensitive technique for detecting glucose uptake into tumours.^20^ CEST MRI techniques can also give a readout of tumour pH (see below)^21^ and Cai et al. have described the potential of these techniques to characterise redox state and NADH concentration.^22^

Magnetic resonance spectroscopy (MRS) allows the non-invasive molecular imaging of many oncometabolites. Most commonly proton MRS (^1^H MRS) has been used to detect known shifts in the resonance of protons that vary with the concentration of specific metabolites in the tumour environment and hence image the metabolic heterogeneity of cancer tissue. In addition, ^13^C MRS is especially suited to measuring metabolic fluxes although this technique typically requires the infusion of ^13^C substrate as natural abundance of ^13^C is limited. Attempts have been made to monitor response to neoadjuvant chemotherapy in breast cancer using choline ^1^H MRS to monitor changes in phospholipid metabolism with limited success.^23^ As described above, gliomas commonly harbour IDH mutations, resulting in very high intratumoural concentrations of 2-HG and presenting a target for MRS imaging. A Phase 1 study using in vivo MRS showed a rapid decrease in 2-HG levels within the tumour following treatment with a novel IDH1 inhibitor, consistent with metabolic response to therapy.^24^ Hyperpolarised ^13^C MRI techniques require the infusion of hyperpolarised ^13^C substrates, most commonly using ^13^C pyruvate although other probes are being developed, and can also allow characterisation of flux through metabolic pathways and signal the presence of cancer.^25^

Window study designs can be used to evaluate novel imaging and understand how the imaging signature reflects tumour biology. We have recently conducted a study, the FRONTIER trial, in which we imaged primary breast cancers with ^18^F-FACBC, shortly prior to tumour sampling at surgical resection (clinicaltrials.gov number: NCT03036943). Here, we plan to correlate immunohistochemical, transcriptomic and metabolomic profiling with ^18^F-FACBC uptake to characterise the metabolic signature for this tracer with a view to understanding its potential to act as a biomarker for drugs targeting cancer metabolism. In a similar manner we are also conducting a study, the IMAGO trial, using Amide CEST-MRI, a non-invasive imaging technique that can determine the pH and presence of hypoxia within the brain.^21^ The CEST and anatomical MRI images are merged to annotate areas of high and low CEST contrast. During neurosurgical resection of the tumour, laser guided biopsies are then taken from areas of high and low CEST contrast and to match tissue-based assays to the imaging signature, again using immunohistochemical, transcriptomic and metabolomic approaches (ISRCTN identifier: ISRCTN86522205).

## Considerations for window trial design

Design of window trials must be undertaken with care and there are particular considerations for cancer metabolism studies. Consistency of sample handling is critical to success and is a particular issue in surgical trials. For example, it has been shown that the proliferation marker Ki67 can significantly vary between samples taken at surgery and ultrasound-guided breast core biopsy.^26^ This is also likely to apply to other sensitive assays such as immunohistochemistry for phosphorylated proteins involved in signalling cascades. Patients undergoing an anaesthetic and major surgery will be undergoing significant physiological stress and often receive glucose infusions that may alter host metabolism. As soon as a tumour is devascularised during resection, the degree of hypoxia will likely increase with profound metabolic consequences. Hence, samples for metabolomic and functional genomic profiling need to be snap frozen at point of biopsy by a dedicated team to optimise quality. Drug PK may inform timing of sampling for example, such that biopsies are taken at point of peak plasma concentration.

Prior to study set up consideration has to be made as to the choice of molecular target to assay and considerable effort should be expended in development of study assays prior to commencing the study. Molecular targets are unlikely to be suitable if there is only limited variability from baseline observed with the intervention of interest in preclinical studies or assay sensitivity is limited. Marked assay replicate variability in assay sampling will require a high magnitude of drug-induced change with regard to the endpoint of interest. A commonly described threshold is that an assay should perform to a level such that an observed 30% change in the molecular signal would be statistically significant.^27^ To this end, the optimisation of immunohistochemical assays often makes use of formalin-fixed cell line pellets manipulated to express the protein of interest at different levels. Once the assay has been optimised in this fashion biobanked human tissue from the tumour site of interest should then be assayed for further validation. Similar approaches apply to other techniques, for example using surrogate tissue samples to optimise metabolite or RNA extraction.

Ethical and pragmatic considerations also apply to study design. Giving a very novel drug to patients with curative disease and undergoing major surgery may not be safe. The window prior to definitive surgery has to be relatively short in order that standard of care treatment is not significantly delayed, and the number of interventions should not be too burdensome for patients. Nuclear medicine interventions using radiolabelled tracers have to be limited in terms of radiation exposure to the patient, particularly in the curative setting.

## Metformin, repurposing of a drug targeting cancer metabolism

Over the past 15 years there has been growing interest in the potential of repurposing the biguanide drug, metformin, as a cancer therapy. This was sparked by a series of epidemiological studies suggesting a decrease in cancer incidence for patients with diabetes that were taking metformin compared with those patients on other anti-hyperglycaemic therapies. This led to the publication of multiple preclinical studies aimed at teasing out the mechanism of anticancer action for biguanide therapy but often with conflicting results. The one consistent theme in these preclinical studies was that the dose of metformin used in order to elicit antineoplastic activity, either in vitro or using animal models, was typically 10–1000 times peak plasma level in patients.^28^ There is strong evidence that metformin inhibits complex 1 of the mitochondrial electron transport chain and two hypotheses have emerged. Firstly, that metformin has a direct anti-mitochondrial effect on tumour cells resulting in reduced tumour growth by (a) limiting carbon flux through the TCA cycle for anabolic metabolism; and (b) disrupting energy metabolism leading to activation of AMP-activated protein kinase (AMPK) and hence a switch from anabolic to catabolic metabolism. Alternatively, other investigators view that its anticancer effects are most probably driven by modulation of host patient metabolism through AMPK activation in hepatocytes decreasing hepatic gluconeogenesis, with subsequently reduced circulating insulin and glucose levels.

This led to a number of clinical ‘window’ studies designed to address whether metformin had any effect on tumour cell proliferation at standard clinical dosing and test preclinical hypotheses as to metformin’s mechanism of action. The first of these published by Hadad et al., recruited 55 patients with early breast cancer and compared tumour tissue taken at biopsy with a later surgical sample and 2 weeks of metformin in between (and also including eight untreated controls). Following metformin treatment there was a significant fall in Ki67 suggestive of an antiproliferative effect and also increased phosphorylation of AMPK consistent with a direct mitochondrial effect. Serum insulin levels remained stable pre- and post-metformin. However, the use of 5% glucose at surgery is likely to have confounded the latter result emphasising how consideration of the peri-operative setting is of critical importance when designing a surgical window study.^29^ Since then three other window studies in breast cancer have been published with conflicting observations with regard to change in Ki67 and/or AMPK activation.^30–32^ One study suggested that effects on proliferation are only observed in an obese subgroup with insulin insensitivity.^30^

Similarly contrasting observations have been observed in window studies assessing metformin in patients with Type 1 endometrial cancer, a disease strongly associated with obesity and Type 2 diabetes. Three early studies have been published all of which demonstrated significant falls in Ki67 and evidence of decreased mTOR signalling.^33–35^ However, a recent larger, double-blind, placebo-controlled, randomised study showed no overall difference in expression of Ki67 or that of markers of the PI3K–mTOR and insulin signalling pathways.^36^ In prostate cancer, a window study recruited 24 patients and a diagnostic core biopsy was compared with a surgical specimen with a significant fall in Ki67 observed following metformin. No direct evidence of AMPK activation was detected but there was a trend toward a decrease in prostate-specific antigen.^37^ Another study in head and neck cancer presented evidence of increased apoptosis and increased CD8^+^ T-cell infiltration following treatment with metformin.^38^

It is not absolutely clear why there was such inconsistency in results from these studies and as such, these results did not move the field much further forward. Recruitment varied between 35 and 200 patients, some with and some without controls, therefore some trials may have been inadequately powered for a highly heterogeneous population. Differences in sample collection at surgery and pre-treatment biopsy may have influenced expression of sensitive immunohistochemical markers including phosphorylated proteins and Ki67, as well as circulating markers of host metabolism. The studies may reflect the limitations of relying on a handful of immunohistochemical biomarkers to characterise drug activity.

A number of studies have also used metabolomic profiling to assess for mitochondrial targeting by metformin. Schuler et al. used this technique in a surgical window study of metformin in endometrial cancer comparing the metabolomic profile of patients dependent on Ki67 response with metformin. Here, they observed an intra-tumoural increase in levels of glucose and glycogen levels, and the ketone body, 3-hydroxybutyrate, in those tumours that had a fall in Ki67 post-metformin. The authors speculated that these changes reflected increased fatty acid oxidation and shunting of glucose to glycogen as a consequence of direct mitochondrial effect.^34^ Although not strictly a window study, Liu et al. also carried out metabolomic profiling of historic ovarian cancer samples taken from patients that had received metformin. They reported reduced levels of a number of mitochondria metabolites compared with untreated controls including TCA intermediates and short-chain acyl carnitines, consistent with mitochondrial interference.^39^

A small pilot study of metformin in healthy patients with Li-Fraumeni syndrome, a cancer predisposition disorder associated with germline p53 mutations, has shown the potential of using other mitochondrial assays in drug studies. Here, Wang et al. showed that the oxygen consumption rate (OCR) was reduced in extracted peripheral blood mononuclear cells. Additionally, there was an increase in the OCR to extracellular acidification rate (ECAR) ratio suggestive of a switch from oxidative to glycolytic metabolism in response to metformin in Li-Fraumeni patients. Skeletal muscle phosphocreatine was also measured using ^31^P magnetic resonance spectroscopy with an increased phosphocreatine recovery time after metformin consistent with a mitochondrial effect.^40^

## Integrating pharmacodynamic assays to characterise metabolic response: a case study

**Fig. 1:**
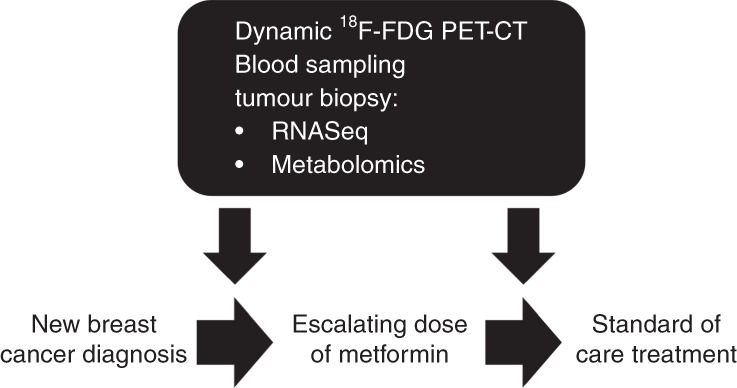
Study schema for a window of opportunity study integrating multiple assays to assay metformin’s effect on breast cancer metabolism. Shortly after diagnosis with primary breast cancer, patients underwent a baseline and post-metformin dynamic ^18^F-FDG PET-CT scan and ultrasound-guided tumour biopsies for mRNA sequencing and metabolomics profiling. Additionally, blood samples were also collected at these timepoints such that metformin’s effect on host metabolism could be related back to intra-tumoural pharmacodynamic changes

We recently conducted a window study, the NEOMET trial, to profile the bioactivity of metformin in breast cancer and characterise heterogeneity of metabolic response.^10^ In this study we recruited 40 women with primary breast cancer prior to definitive therapy and carried out a series of pharmacodynamic assays either side of a 2-week window of metformin, including novel imaging, ultrasound guided core biopsies of the primary tumour and blood sampling to characterise changes in host metabolism (see Fig. 1 for study design).

We utilised functional imaging with dynamic ^18^F-FDG PET-CT to identify subtle changes in glucose uptake into the primary tumour. In contrast to standard clinical static PET-CT imaging, dynamic acquisition requires multiple images taken over a specified time period, in this case 45 min. This allows for kinetic analysis of tumour FDG uptake generating a flux constant (K_FDG_) that describes the rate of intra-cellular FDG phosphorylation. Additionally, by using an input function, in this case from aortic blood flow, dynamic imaging adjusts for any metformin-induced changes in circulating blood glucose. Using this sensitive methodology, we observed a significant increase in K_FDG_ following metformin treatment but no change in the standard static ^18^F-FDG uptake measures SULmax and SULmean (standardised uptake values normalised for lean body mass).

We then went on to carry out mRNA sequencing of the tumour samples pre- and post-metformin and identified multiple mitochondrial pathways that were upregulated at the transcriptomic level. Additionally, metabolomic profiling of the samples demonstrated decreases in the levels of several mitochondrial metabolites including two short-chain acyl-carnitines consistent with findings from another metabolomic study of tumour samples from metformin-treated patients.^39^ In summary, the observed increase in glucose uptake and transcriptomic and metabolomic response patterns were consistent with metformin targeting mitochondria. By integrating these datasets, we were then able to observe two distinct metabolic responses; (a) an OXPHOS transcriptional response (OTR) group for which there is an increase in OXPHOS gene transcription and decreased short-chain acyl-carnitine levels, and (b) an FDG response group with increased ^18^F-FDG uptake. Ongoing work is aimed at identifying baseline biomarkers that can discriminate between these two groups. This case study describes how the integration of multiple assays including novel molecular imaging, transcriptomics and metabolomics can provide, for drugs that target cancer metabolism, substantial insight into the nature of target engagement, selection of patients and mechanisms of resistance.

Further work is ongoing and, based on this initial characterisation of dynamic response to metformin, we plan to assay for baseline markers of interest with a view to informing future stratification in late-phase clinical trials. For example, prior preclinical analysis has suggested that mutations in mtDNA encoding for mitochondrial complex 1 may define metabolic response to biguanide therapy.^41^ Hence, limited sequencing of mtDNA from banked tumour samples is being carried out to explore this hypothesis.

## Summary

Window study designs can enable detailed characterisation of the pharmacodynamic effects of drugs or the tumour biology that lies behind an imaging signature. Such studies may occur in the setting of the short window prior to instigation of standard therapy just after diagnosis offering a unique opportunity to study pharmacodynamic response in the setting of a tumour unperturbed by prior therapy. Modern molecular imaging, and metabolomic and functional genomic techniques, can now be applied to pharmacodynamic cancer metabolism studies, and are powerful tools to identify target engagement and understand resistance to therapy. However, it is important to be attentive to window trial design as flaws in sample handling, choice of assay and insufficient power will compromise a study.

## Data Availability

Not applicable
